# Supplementary material for: Organophosphorus Flame Retardants: A Global Review of Indoor Contamination and Human Exposure in Europe and Epidemiological Evidence
Source: Int J Environ Res Public Health. 2020 Sep 15;17(18):6713. doi: 10.3390/ijerph17186713 (PMC7558007; doi:10.3390/ijerph17186713)
Supplement: Supplementary file 1 [file ijerph-17-06713-s001.pdf]

International Journal of
Environmental Research and Public Health

MDPI

Supplementary Table S1. OPFRs abbreviation.

CAS Number	Used Acronyme	Commonly Used Acronyme	International Name	Metabolite Used Acronyme	Metabolite Commonly Used Acronyme	International Name	Others Metabolite
38051-10-4	BCMP-BCEP	V6, TCDDP	Tetrakis (2-chlorethyl) dichloroisopentyl diphosphate				
5945-33-5	BDP	BADP, BAPP, BPADP, BPA-BDPP	Bisphenol-A diphenyl phosphate				
2752-95-6	BdPhP		Butyl diphenyl phosphate				
2528-36-1	dBPhP		Dibutyl phenyl phosphate				
838-85-7	DPP	DPK	Diphenylphosphate				
1241-94-7	EHDPP	EDP, EHDP, EHDPP,DPEHP, EHDPHP	Ethylhexyldiphényl phosphate				5-HO-EHDPHP, EHPHP
29761-21-5	iDDPHP	IDDP,IDPP	Isodecyldiphenylphosphate				
249-828-6	iDPP		Isodecyl diphenyl phosphate				
	ITP	IPTP, IPTPP	Isopropylated triarylphosphate esters	IP-PPP		Isopropylphenyle phenyl phosphate	tb-PPP
57583-54-7	PBDPP	RDP	Resorcinol-bis (diphenyl) phosphate				
78-51-3	TBOEP	TBEP, TBXP	Tris (2-butoxyethyl) phosphate	BBOEP		Bis(2-butoxyethyl) phosphate	3-HO-TBEP
				BBOEHEP		Bis(2-butoxyethyl)-(2-hydroxyethyl) phosphate	
				DBOEP	DBEP	Di-(2-butoxyethyl) phosphate	
78-33-1	TBPP	TBDP	Tris (p-t-butylphenyl) phosphate				
115-96-8	TCEP		Tris (2-chloroethyl) phosphate	BCEP		Bis-(2-chlorethyl)-phosphate	uTCEP
				DCEP		Di-(2-chloroethyl) phosphate	
13674-84-5	TCIPP	TCPP, TCiPP	Dris(2-chloroisopropyl)phosphate	BCIPP	BCPP	Bis(1-chloro-2-propyl) phosphate	BCIPHIPP
				DCIPP	DCPP	Di-(2-chloroisopropyl) phosphate	
126-72-7	TDBPP	TDBP, TDB99	Tris (2,3-Dibromopropyl) phosphate				
13674-87-8	TDCIPP	TDCPP, TDCiPP	Tris(1,3-dichloro-2-propyl) phosphate	BDCIPP	BDCPP	Bis(1,3-dichloro-2-propyl) phosphate	
78-42-2	TEHP	TOF, TOP	Tris (2-ethylhexyl) phosphate	DEHP		Diethylhexyl phosphate	BEHP
78-40-0	TEP		Triethyphosphate	DEP		Diethyl phosphate	
2528-39-4	THP		Trihexyl phosphate				
2528-39-4	THP		Trihexyl phosphate				
126-71-6	TIBP	TiBP, TIP	Tri-iso-butylphosphate				
513-02-0	TIPP	TIPrP, TiPP, TiPrP	Tri-isopropylphosphate				
64532-95-2	TiPPP	o-IPP, T2IPPP	Phenol isopropylated phosphate, ortho				
563-04-2	TmCP		Tri-m-cresylphosphate	DmCP		Di-m-cresyl phosphate	
512-56-1	TMP	TMPA, TMPO	Trimethylphosphate				

1330-78-5	TMPP	TCrP, TTOP, TTP, TBT, TCP, TBT	Tricresylphosphate (mixture o, m, p)				DMPP
126-73-8	TNBP	TnBP, TBP, TB	Tri-n-butylphosphate	DnBP	DBP	Di-n-butyl phosphate	
78-30-8	ToCP	TOCP,TOTP,o-TCP, o-TMPP	Tri-o-cresylphosphate	DoCP		Di-o-cresyl phosphate	
78-32-0	TpCP	TPCP,p-TCP	Tri-p-cresylphosphate	DpCP		Di-p-cresyl phosphate	
2528-38-3	TPEP		Tripentylphosphate				
115-86-6	TPHP	TPP, TPhP	Triphenylphosphate	DPHP	DPP	Diphenyl phosphate	4-HO-DPHP, 3-HO-TPHP, 4-HO-TPHP
513-08-6	TPP	TnPP, TPrP, TPPO,TnPrP	Tri-n-propylphosphate				
25653-16-1	TXP	TDMPP	Tris(3,5-xylenyl)phosphate				

Supplementary Table S2. Concentrations of OPFRs in indoor dust (μg/g), Europe, 2000-2018.

CAS number	Abbreviation	Period	Country	Sampling Location	Sample Size	Min.	Max.	Mean	Median	References
38051-10-4	BCMP-BCEP	2013	UK	Homes	10	0	0.76	0.1	0.02	Kademoglou et al.,2017
		2013–2014	Norway	Homes	10	0	0.01	0	0	Kademoglou et al.,2017
		-	Greece	Cars	25	0	8.54	0.68	0.01 *	Christia et al.,2018
5945-33-5	BDP	2013	UK	Homes	10	<0.0034	0.49	0.12	0.07	Kademoglou et al.,2017
		2013–2014	Norway	Homes	10	<0.0034	0.7	0.12	-	Kademoglou et al.,2017
		-	Greece	Cars	25	0	0.42	0.04	0.02 *	Christia et al.,2018
838-85-7	DPP	2016	The netherlands	Homes (on top of electronics)	11	0.22	6.59	2.78	2.58	Björnsdotter et al., 2018
		2016	The netherlands	Homes and Offices	12	0.15	4.19	1.2	0.74	Björnsdotter et al., 2018
		2017	Spain	Cars	15	0.92	79.66	8.29	2.31	Björnsdotter et al., 2018
		2017	Spain	Homes (on top of electronics)	13	0.3	21.9	3.21	1.75	Björnsdotter et al., 2018
1241-94-7	EHDPP	2008	Danmark (Odense)	Daycare centers (shelves,ledges,windows sills)	151	<0.14	540	-	2.1	Langer et al.,2016
		2008	Danmark (Odense)	Homes (children bedroom's,shelves,ledges,windows sills)	497	<0.14	11	-	<014	Langer et al.,2016
		2008	Swenden (Stockolm)	Homes	62	<0.23	20	-	2.7	Luongo and Östman.,2016
		2010–2011	Portugal	Homes	28	0.15	7.2	1.1	0.62	Coelho et al.,2016
		2011–2012	Germany	Dayecare centers	63	0.3	95.6	4.13	1.1	Fromme et al.,2014
		2011–2012	Germany	Cars	19	0.01	1.8	-	1.2	Harrad et al.,2016
		2011–2012	Germany	Homes (living rooms)	22	<0.01	0.56	-	0.14	Harrad et al.,2016
		2011–2012	Germany	Offices	25	0.13	3.8	-	0.36	Harrad et al.,2016
		2011–2012	UK (West Midlands)	Cars	21	0.29	11	2.9	2.2	Brommer and Harrad.,2015
		2011–2012	UK (West Midlands)	Classroom	28	0.3	470	50	29	Brommer and Harrad.,2015
		2011–2012	UK (West Midlands)	Homes (living room)	32	0.18	130	2.6	1.6	Brommer and Harrad.,2015
		2011–2012	UK (West Midlands)	Offices	61	0.15	0.05	81	10	Brommer and Harrad.,2015
		2012	Norway	Homes	48	-	5.9	0.88	0.62	Cequier et al.,2014
		2013	Czech Republic	Homes	30	0.14	8.23	-	0.84	Vykoukalová et al.,2017
		2013	UK	Homes	10	0.29	9.17	3.01	2.38	Kademoglou et al.,2017
		2013–2014	Norway	Homes	10	0.04	4.01	0.74	0.2	Kademoglou et al.,2017
		2013–2014	Norway (Oslo)	Homes (floor)	61	0.083	12.5	-	0.42	Xu et al.,2016
		2013–2014	Norway (Oslo)	Homes (surface)	61	0.212	30.2	-	0.455	Xu et al.,2016
		-	Sweden (Stockolm)	Daycare center	10	0.2	160	24	0.8	Bergh et al.,2011

		-	Sweden (Stockolm)	Homes	10	N.D	1.8	0.6	0.5	Bergh et al.,2011
		-	Sweden (Stockolm)	Offices	10	N.D	73	15	1	Bergh et al.,2011
29761-21-5	iDDPHP	-	Greece	Cars	25	0.04	4.63	0.36	0.12 *	Christia et al.,2018
249-828-6	iDPP	2013	UK	Homes	10	0.11	1.69	0.62	0.4	Kademoglou et al.,2017
		2013–2014	Norway	Homes	10	0.01	0.26	0.08	0.05	Kademoglou et al.,2017
57583-54-7	PBDPP	2013	UK	Homes	10	<0.0018	0	0	0	Kademoglou et al.,2017
		2013–2014	Norway	Homes	10	<0.0018	<0.0018	<0.0018	<0.0018	Kademoglou et al.,2017
		-	Greece	Cars	25	0	0.65	0.04	0.004 *	Christia et al.,2018
78-51-3	TBOEP	2008	Swenden (Stockolm)	Homes	62	<0.20	107	-	11	Luongo and Östman.,2016
		2008	Belgium	Homes (floor)	33	0.2	55.7	11.8	3.61	Van den Eede et al.,2011
		2008	Belgium	Shop	15	0.2	55.7	11.8	3.61	Van den Eede et al.,2011
		2008	Danmark (Odense)	Daycare centers (shelves,ledges,windows sills)	151	<0.40	11000	-	26	Langer et al.,2016
		2008	Danmark (Odense)	Homes (children bedroom's,shelves,ledges,windows sills)	497	<0.40	1300	-	<0.40	Langer et al.,2016
		2010	Roumania (Lasi)	Homes	47	<0.05	21	2.7	1.5	Dirtu et al.,2012
		2010–2011	Germany	Cars	12	<0.06	74	21	7.5	Brommer et al.,2012
		2010–2011	Germany	Offices	10	2.9	13	7	-	Brommer et al.,2012
		2011–2012	Germany	Dayecare centers	63	1.61	4711	706	225	Fromme et al.,2014
		2012	Norway	Homes	48	-	128	18.7	13.4	Cequier et al.,2014
		2013	UK	Homes	10	0.23	58.75	16.08	8.07	Kademoglou et al.,2017
		2013–2014	Norway	Homes	10	1.34	48.01	19.15	18.36	Kademoglou et al.,2017
		2013–2014	Norway (Oslo)	Homes (floor)	61	0.73	311	-	8.15	Xu et al.,2016
		2013–2014	Norway (Oslo)	Homes (surface)	61	<0.090	540	-	6.8	Xu et al.,2016
		2015	Germany (Rhine/Main area)	Cars	11	2.3	200	35	7.5	Zhou et al.,2017
		2015	Germany (Rhine/Main area)	Homes	15	2.1	99	13	4.3	Zhou et al.,2017
		2015	Germany (Rhine/Main area)	Offices	11	4.8	150	45	8.6	Zhou et al.,2017
		-	Greece	Cars	25	0.19	9.78	1.41	0.90 *	Christia et al.,2018
		-	Sweden (Stockolm)	Daycare center	10	31	4100	1900	1600	Bergh et al.,2011
		-	Sweden (Stockolm)	Homes	10	0.6	30	8.5	4	Bergh et al.,2011
		-	Sweden (Stockolm)	Offices	10	4.5	960	250	87	Bergh et al.,2011
78-33-1	TBPP	2013	Czech Republic	Homes	30	0	0.04	-	0.01	Vykoukalová et al.,2017
115-96-8	TCEP	2005	Austria	Schools	34	0.6	35	-	2.5	Huuter et al.,2013
		2008	Danmark (Odense)	Daycare centers (shelves,ledges,windows sills)	151	<0.6	1800	-	16	Langer et al.,2016
		2008	Danmark (Odense)	Homes (children bedroom's,shelves,ledges,windows sills)	497	<0.60	230	-	6.9	Langer et al.,2016
		2008	Swenden (Stockolm)	Homes	62	<0.19	808	-	4	Luongo and Östman.,2016
		2008	Belgium	Homes (floor)	33	<0.08	2.65	0.49	0.23	Van den Eede et al.,2011
		2008	Belgium	Shop	15	<0.08	5.46	1.17	0.59	Van den Eede et al.,2011
		2010	Roumania (Lasi)	Homes	47	<0.02	1.16	0.18	0.1	Dirtu et al.,2012
		2010–2011	Germany	Cars	12	<0.08	5.8	0.95	0.28	Brommer et al.,2012
		2010–2011	Germany	Offices	10	<0.08	0.17	0.12	-	Brommer et al.,2012
		2010–2011	Portugal	Homes	28	<0.004	0.72	0.06	0.02	Coelho et al.,2016
		2011–2012	UK (West Midlands)	Cars	21	<0.06	8.7	1.95	1.23	Brommer and Harrad.,2015
		2011–2012	UK (West Midlands)	Classroom	28	<0.06	8.3	1.5	0.86	Brommer and Harrad.,2015
		2011–2012	UK (West Midlands)	Homes (living room)	32	<0.06	28	2.2	0.81	Brommer and Harrad.,2015

		2011–2012	UK (West Midlands)	Offices	61	<0.06	160	5	0.87	Brommer and Harrad.,2015
		2011–2012	Germany	Daycare centers	63	0.1	8.3	1.35	0.4	Fromme et al.,2014
		2011–2012	Germany	Cars	19	<0.06	5.1	-	0.4	Harrad et al.,2016
		2011–2012	Germany	Homes (living rooms)	22	<0.06	5.7	-	0.21	Harrad et al.,2016
		2011–2012	Germany	Offices	25	<0.06	12	-	0.13	Harrad et al.,2016
		2012	Norway	Homes	48	-	4.63	0.8	0.41	Cequier et al.,2014
		2013	UK	Homes	10	0.14	6.27	1.57	0.87	Kademoglou et al.,2017
		2013	Czech Republic	Homes	30	0.04	1.68	-	0.16	Vykoukalová et al.,2017
		2013–2014	Norway	Homes	10	0.06	0.5	0.74	0.2	Kademoglou et al.,2017
		2013–2014	Norway (Oslo)	Homes (floor)	61	<0.170	350	-	0.435	Xu et al.,2016
		2013–2014	Norway (Oslo)	Homes (surface)	61	<0.170	15.2	-	0.455	Xu et al.,2016
		2013–2015	Netherlands	Homes (playroom)	50	0.02	5.66	-	0.16	Sugeng et al.2018
		2015	Germany (Rhine/Main area)	Cars	11	<LOD	170	18	1.4	Zhou et al.,2017
		2015	Germany (Rhine/Main area)	Homes	15	<LOD	5	1.3	1.1	Zhou et al.,2017
		2015	Germany (Rhine/Main area)	Offices	11	0.93	18	3.7	1.9	Zhou et al.,2017
		-	Sweden (Stockolm)	Daycare center	10	2.5	150	51	30	Bergh et al.,2011
		-	Sweden (Stockolm)	Homes	10	N.D	33	7.6	2.1	Bergh et al.,2011
		-	Sweden (Stockolm)	Offices	10	1.3	260	36	6.7	Bergh et al.,2011
		-	Greece	Cars	25	0.01	13.14	1.24	0.11 *	Christia et al.,2018
13674-84-5	TCIPP	2008	Belgium	Homes (floor)	33	0.19	73.7	4.82	1.38	Van den Eede et al.,2011
		2008	Belgium	Shop	15	0.58	24.4	5.16	2.94	Van den Eede et al.,2011
		2008	Danmark (Odense)	Daycare centers (shelves,ledges,windows sills)	151	<1.10	350	-	5.6	Langer et al.,2016
		2008	Danmark (Odense)	Homes (children bedroom's,shelves,ledges,windows sills)	497	<1.10	100	-	<1,10	Langer et al.,2016
		2008	Swenden (Stockolm)	Homes	62	1.21	98	-	11	Luongo and Östman.,2016
		2010	Roumania (Lasi)	Homes	47	<0.02	16.4	2.5	0.86	Dirtu et al.,2012
		2010–2011	Germany	Cars	12	1.4	4.3	3.1	3.2	Brommer et al.,2012
		2010–2011	Germany	Offices	10	0.18	9.4	3	-	Brommer et al.,2012
		2011–2012	Germany	Dayecare centers	63	0.71	47	4.65	2.68	Fromme et al.,2014
		2011–2012	Germany	Cars	19	0.29	100	-	2.9	Harrad et al.,2016
		2011–2012	Germany	Homes (living rooms)	22	0.33	5.7	-	1	Harrad et al.,2016
		2011–2012	Germany	Offices	25	0.18	13	-	1.6	Harrad et al.,2016
		2011–2012	UK (West Midlands)	Cars	21	2.4	370	83	53	Brommer and Harrad.,2015
		2011–2012	UK (West Midlands)	Classroom	28	1.7	210	33	16	Brommer and Harrad.,2015
		2011–2012	UK (West Midlands)	Homes (living room)	32	3.7	100	29	21	Brommer and Harrad.,2015
		2011–2012	UK (West Midlands)	Offices	61	3.6	230	44	33	Brommer and Harrad.,2015
		2012	Norway	Homes	48	-	40.1	5.74	2.68	Cequier et al.,2014
		2013	Czech Republic	Homes	30	0.16	26.7	-	1.86	Vykoukalová et al.,2017
		2013–2014	Norway (Oslo)	Homes (surface)	61	<0.055	498	-	5.24	Xu et al.,2016
		2013–2014	Norway (Oslo)	Homes (floor)	61	<0.055	145	-	2	Xu et al.,2016
		2013–2015	Netherlands	Homes (playroom)	50	0.14	0.03	-	0.83	Sugeng et al.2018
		2015	Germany (Rhine/Main area)	Cars	11	4	340	41	7.8	Zhou et al.,2017
		2015	Germany (Rhine/Main area)	Homes	15	1.7	10	5	4.2	Zhou et al.,2017
		2015	Germany (Rhine/Main area)	Offices	11	1.8	470	52	8.5	Zhou et al.,2017

		-	Greece	Cars	25	0.11	101.8	8.06	2.61 *	Christia et al.,2018
		-	Sweden (Stockolm)	Daycare center	10	0.8	12	4.5	3.1	Bergh et al.,2011
		-	Sweden (Stockolm)	Homes	10	0.7	11	3.1	1.6	Bergh et al.,2011
		-	Sweden (Stockolm)	Offices	10	3.4	120	32	19	Bergh et al.,2011
126-72-7	TDBPP	2013	UK	Homes	10	<0.0005	0	0	<0,0005	Kademoglou et al.,2017
		2013	Czech Republic	Homes	30	N.D	N.D	-	N.D	Vykoukalová et al.,2017
		2013–2014	Norway	Homes	10	0	0.02	0.01	0	Kademoglou et al.,2017
13674-87-8	TDCIPP	2008	Belgium	Homes (floor)	33	<0.08	6.64	0.57	0.36	Van den Eede et al.,2011
		2008	Belgium	Shop	15	<0.08	56.2	4.61	0.76	Van den Eede et al.,2011
		2008	Danmark (Odense)	Daycare centers (shelves,ledges,windows sills)	151	<1.20	320	-	7.1	Langer et al.,2016
		2008	Danmark (Odense)	Homes (children bedroom's,shelves,ledges,windows sills)	497	<1.20	860	-	<1,20	Langer et al.,2016
		2008	Swenden (Stockolm)	Homes	62	<0.60	12	-	2	Luongo and Östman.,2016
		2010	Roumania (Lasi)	Homes	47	<0.02	0.46	0.1	0.06	Dirtu et al.,2012
		2010–2011	Germany	Cars	12	<0.08	620	130	21	Brommer et al.,2012
		2010–2011	Germany	Offices	10	<0.08	0.29	0.15	-	Brommer et al.,2012
		2010–2011	Portugal	Homes	28	<0.0018	0.39	0.06	0.02	Coelho et al.,2016
		2011–2012	Germany	Cars	19	<0.03	620	-	4.1	Harrad et al.,2016
		2011–2012	Germany	Homes (living rooms)	22	<0.03	14	-	0.08	Harrad et al.,2016
		2011–2012	Germany	Offices	25	<0.03	2.2	-	0.14	Harrad et al.,2016
		2011–2012	UK (West Midlands)	Cars	21	0.11	740	110	31	Brommer and Harrad.,2015
		2011–2012	UK (West Midlands)	Classroom	28	0.04	10	1.1	0.51	Brommer and Harrad.,2015
		2011–2012	UK (West Midlands)	Homes (living room)	32	0.06	14	2	0.71	Brommer and Harrad.,2015
		2011–2012	UK (West Midlands)	Offices	61	<0.03	51	2.1	0.48	Brommer and Harrad.,2015
		2012	Norway	Homes	48	-	6.92	0.8	0.5	Cequier et al.,2014
		2013	Czech Republic	Homes	30	0.06	1.22	-	0.18	Vykoukalová et al.,2017
		2013	UK	Homes	10	0.35	3.79	1.08	0.75	Kademoglou et al.,2017
		2013–2014	Norway	Homes	10	0.08	2.31	0.51	0.34	Kademoglou et al.,2017
		2013–2014	Norway (Oslo)	Homes (floor)	61	<0.055	6	-	0.4	Xu et al.,2016
		2013–2014	Norway (Oslo)	Homes (surface)	61	<0.055	366	-	1.13	Xu et al.,2016
		2013–2015	Netherlands	Homes (playroom)	50	0.03	0.01	-	0.7	Sugeng et al.2018
		2015	Germany (Rhine/Main area)	Cars	11	3	56	16	4.7	Zhou et al.,2017
		2015	Germany (Rhine/Main area)	Homes	15	<LOD	4.3	0.85	<LOD	Zhou et al.,2017
		2015	Germany (Rhine/Main area)	Offices	11	0.8	39	6.3	3.2	Zhou et al.,2017
		-	Greece	Cars	25	0.02	85.04	7.52	2.51 *	Christia et al.,2018
		-	Sweden (Stockolm)	Daycare center	10	3.9	150	28	9.1	Bergh et al.,2011
		-	Sweden (Stockolm)	Homes	10	2.2	27	12	10	Bergh et al.,2011
		-	Sweden (Stockolm)	Offices	10	3.3	91	30	17	Bergh et al.,2011
78-42-2	TEHP	2008	Danmark (Odense)	Daycare centers (shelves,ledges,windows sills)	151	<2.50	3.8	-	<2.50	Langer et al.,2016
		2008	Danmark (Odense)	Homes (children bedroom's,shelves,ledges,windows sills)	497	<2.50	11	-	<2.50	Langer et al.,2016
		2008	Swenden (Stockolm)	Homes	62	<2.10	46	-	<2.10	Luongo and Östman.,2016
		2010–2011	Portugal	Homes	28	0.25	2.3	2.2	1.7	Coelho et al.,2016
		2011–2012	Germany	Daycare centers	63	<0.30	4.85	0.68	0.5	Fromme et al.,2014
		2013	Czech Republic	Homes	30	0.04	10.3	-	0.15	Vykoukalová et al.,2017

		2013	UK	Homes	10	0.1	0.47	0.22	0.16	Kademoglou et al.,2017
		2013–2014	Norway	Homes	10	0.11	0.62	0.24	0.18	Kademoglou et al.,2017
		2013–2014	Norway (Oslo)	Homes (floor)	61	<0.140	3.98	-	0.4	Xu et al.,2016
		2013–2014	Norway (Oslo)	Homes (surface)	61	<0.140	450	-	0.71	Xu et al.,2016
		2015	Germany (Rhine/Main area)	Cars	11	0.7	14	3.1	2	Zhou et al.,2017
		2015	Germany (Rhine/Main area)	Homes	15	<LOD	2.1	0.14	<LOD	Zhou et al.,2017
		2015	Germany (Rhine/Main area)	Offices	11	<LOD	1.4	0.43	<LOD	Zhou et al.,2017
		-	Sweden (Stockolm)	Daycare center	10	N.D	0.7	0.2	0.1	Bergh et al.,2011
		-	Sweden (Stockolm)	Homes	10	N.D	0.2	N.D	N.D	Bergh et al.,2011
		-	Sweden (Stockolm)	Offices	10	N.D	0.3	0.1	N.D	Bergh et al.,2011
78-40-0	TEP	2008	Belgium	Homes (floor)	33	N.D	N.D	<0.05	<0.05	Van den Eede et al.,2011
		2008	Belgium	Shop	15	<0.05	0.37	0.13	0.06	Van den Eede et al.,2011
		2008	Swenden (Stockolm)	Homes	62	<0.03	4.3	-	0.19	Luongo and Östman.,2016
		2010	Romania (Lasi)	Homes	47	<0.01	0.42	0.03	0.02	Dirtu et al.,2012
		2015	Germany (Rhine/Main area)	Cars	11	0.2	4.2	0.99	0.44	Zhou et al.,2017
		2015	Germany (Rhine/Main area)	Homes	15	<LOD	2.3	0.31	<LOD	Zhou et al.,2017
		2015	Germany (Rhine/Main area)	Offices	11	0.25	4.3	0.82	0.42	Zhou et al.,2017
		-	Sweden (Stockolm)	Daycare center	10	N.D	4.7	0.7	0.2	Bergh et al.,2011
		-	Sweden (Stockolm)	Homes	10	N.D	N.D	N.D	N.D	Bergh et al.,2011
		-	Sweden (Stockolm)	Offices	10	N.D	0.3	0.1	0.1	Bergh et al.,2011
2528-39-4	THP	2008	Swenden (Stockolm)	Homes	62	<0.03	5.8	-	<0.30	Luongo and Östman.,2016
126-71-6	TIBP	2008	Belgium	Homes (floor)	33	0.7	15.6	4.2	2.99	Van den Eede et al.,2011
		2008	Danmark (Odense)	Daycare centers (shelves,ledges,windows sills)	151	<0.03	2.5	-	<0.03	Langer et al.,2016
		2008	Danmark (Odense)	Homes (children bedroom's,shelves,ledges,windows sills)	497	<0.03	37	-	<0,03	Langer et al.,2016
		2008	Swenden (Stockolm)	Homes	62	<0.02	47	-	5.3	Luongo and Östman.,2016
		2010	Roumania (Lasi)	Homes	47	<0.10	2.39	0.4	0.39	Dirtu et al.,2012
		2011–2012	Germany	Daycare centers	63	<0.30	8.3	0.77	<0.30	Fromme et al.,2014
		2015	Germany (Rhine/Main area)	Cars	11	0.28	2.3	0.75	0.51	Zhou et al.,2017
		2015	Germany (Rhine/Main area)	Homes	15	<LOD	0.82	0.37	0.38	Zhou et al.,2017
		2015	Germany (Rhine/Main area)	Offices	11	0.31	1.3	0.68	0.66	Zhou et al.,2017
		-	Sweden (Stockolm)	Daycare center	10	0.5	1.5	0.9	0.7	Bergh et al.,2011
		-	Sweden (Stockolm)	Homes	10	0.4	3.6	1.4	1.1	Bergh et al.,2011
		-	Sweden (Stockolm)	Offices	10	0.5	9.2	2.4	1.3	Bergh et al.,2011
513-02-0	TIPP	2008	Swenden (Stockolm)	Homes	62	<0.02	0.4	-	<0.02	Luongo and Östman.,2016
64532-95-2	TiPPP	2013	Czech Republic	Homes	30	0	0.51	-	0.01	Vykoukalová et al.,2017
1330-78-5	TMPP	2008	Belgium	Homes (floor)	33	<0.04	5.07	0.44	0.24	Van den Eede et al.,2011
		2008	Belgium	Shop	15	<0.04	12.5	1.53	0.2	Van den Eede et al.,2011
		2008	Danmark (Odense)	Daycare centers (shelves,ledges,windows sills)	151	<0.89	36	-	<0.89	Langer et al.,2016
		2008	Danmark (Odense)	Homes (children bedroom's,shelves,ledges,windows sills)	497	<0.89	18	-	<0.89	Langer et al.,2016
		2008	Swenden (Stockolm)	Homes	62	<0.49	31	-	2.7	Luongo and Östman.,2016
		2010	Roumania (Lasi)	Homes	47	<0.05	5.5	1	0.5	Dirtu et al.,2012
		2010–2011	Portugal	Homes	28	0.02	0.48	0.13	0.1	Coelho et al.,2016
		2011–2012	Germany	Cars	12	<0.04	150	24	1.2	Brommer et al.,2012

		2011–2012	Germany	Offices	10	<0.04	1.9	0.37	-	Brommer et al.,2012
		2011–2012	Germany	Cars	19	<0.01	150	-	0.86	Harrad et al.,2016
		2011–2012	Germany	Homes (living rooms)	22	<0.01	1.3	-	0.14	Harrad et al.,2016
		2011–2012	Germany	Offices	25	<0.01	1.9	-	1.9	Harrad et al.,2016
		2011–2012	UK (West Midlands)	Cars	21	<0.01	5.6	1	0.59	Brommer and Harrad.,2015
		2011–2012	UK (West Midlands)	Classroom	28	<0.01	5.8	0.05	<0.01	Brommer and Harrad.,2015
		2011–2012	UK (West Midlands)	Homes (living room)	32	<0.01	14	2	0.02	Brommer and Harrad.,2015
		2011–2012	UK (West Midlands)	Offices	61	<0.01	5.3	0.33	<0.01	Brommer and Harrad.,2015
		2012	Norway	Homes	48	-	16.2	1.12	0.31	Cequier et al.,2014
		2013	UK	Homes	10	0.08	1.05	0.46	0.29	Kademoglou et al.,2017
		2013–2014	Norway	Homes	10	0.11	3.18	0.5	0.19	Kademoglou et al.,2017
		2013–2014	Norway (Oslo)	Homes (dust)	61	<0.020	30.1	-	0.33	Xu et al.,2016
		2013–2014	Norway (Oslo)	Homes (floor)	61	<0.020	7.52	-	0.18	Xu et al.,2016
		2013–2015	Netherlands	Homes (playroom)	50	0	3.99		0.21	Sugeng et al.2018
		2015	Germany (Rhine/Main area)	Cars	11	<LOD	120	14	2.3	Zhou et al.,2017
		2015	Germany (Rhine/Main area)	Homes	15	<LOD	3.3	0.43	<LOD	Zhou et al.,2017
		2015	Germany (Rhine/Main area)	Offices	11	<LOD	48	8.2	0.34	Zhou et al.,2017
		-	Greece	Cars	25	0.02	0.88	0.15	0.03 *	Christia et al.,2018
		-	Sweden (Stockolm)	Daycare center	10	N.D	13	2	0.4	Bergh et al.,2011
		-	Sweden (Stockolm)	Homes	10	N.D	3	1.2	1	Bergh et al.,2011
		-	Sweden (Stockolm)	Offices	10	N.D	2.9	0.8	0.6	Bergh et al.,2011
126-73-8	TNBP	2008	Belgium	Homes (floor)	33	0.03	2.7	0.25	0.13	Van den Eede et al.,2011
		2008	Belgium	Shop	15	0.05	6.01	0.63	0.21	Van den Eede et al.,2011
		2008	Danmark (Odense)	Daycare centers (shelves,ledges,windows sills)	151	<0.11	4.3	-	<0,11	Langer et al.,2016
		2008	Danmark (Odense)	Homes (children bedroom's,shelves,ledges,windows sills)	497	<0.11	4	-	<0,11	Langer et al.,2016
		2008	Swenden (Stockolm)	Homes	62	0.12	162	-	5.6	Luongo and Östman.,2016
		2010	Roumania (Lasi)	Homes	47	<0.02	0.38	0.07	0.05	Dirtu et al.,2012
		2011–2012	Germany	Cars	12	<0.03	0.63	0.11	0.02	Brommer et al.,2012
		2011–2012	Germany	Offices	10	<0.03	0.41	0.22	-	Brommer et al.,2012
		2011–2012	Portugal	Homes	28	<0.00044	0.55	0.07	0.03	Coelho et al.,2016
		2011–2012	Germany	Daycare centers	63	<0.30	3.7	0.29	<0.30	Fromme et al.,2014
		2011–2012	Germany	Cars	19	<0.03	0.63	-	<0.03	Harrad et al.,2016
		2011–2012	Germany	Homes (living rooms)	22	<0.03	0.25	-	<0.03	Harrad et al.,2016
		2011–2012	Germany	Offices	25	<0.03	0.76	-	0.17	Harrad et al.,2016
		2011–2012	UK (West Midlands)	Cars	21	<0.03	1.2	0.14	<0.03	Brommer and Harrad,.2015
		2011–2012	UK (West Midlands)	Classroom	28	<0.03	0.46	0.17	0.12	Brommer and Harrad,.2015
		2011–2012	UK (West Midlands)	Homes (living room)	32	<0.03	0.09	0.04	<0.03	Brommer and Harrad,.2015
		2011–2012	UK (West Midlands)	Offices	61	<0.03	1.3	0.1	<0.03	Brommer and Harrad,.2015
		2012	Norway	Homes	48	-	0.98	0.12	0.06	Cequier et al.,2014
		2013	Czech Republic	Homes	30	0.01	0.16	-	0.05	Vykoukalová et al.,2017
		2013	UK	Homes	10	0.21	0.48	0.31	0.26	Kademoglou et al.,2017
		2013–2014	Norway	Homes	10	0.23	3.12	0.71	0.49	Kademoglou et al.,2017
		2013–2014	Norway (Oslo)	Homes (floor)	61	<0.190	2.95	-	<0.190	Xu et al.,2016

		2013–2014	Norway (Oslo)	Homes (surface)	61	<0.190	3.03	-	<0.190	Xu et al.,2016
		2015	Germany (Rhine/Main area)	Cars	11	0.21	1.3	0.62	0.41	Zhou et al.,2017
		2015	Germany (Rhine/Main area)	Homes	15	<LOD	1.4	0.32	1.4	Zhou et al.,2017
		2015	Germany (Rhine/Main area)	Offices	11	<LOD	1.2	0.38	0.28	Zhou et al.,2017
		-	Sweden (Stockolm)	Daycare center	10	0.1	6.2	2	1.2	Bergh et al.,2011
		-	Sweden (Stockolm)	Homes	10	N.D	1.7	0.6	0.3	Bergh et al.,2011
		-	Sweden (Stockolm)	Offices	10	N.D	3.2	0.7	0.2	Bergh et al.,2011
78-30-8	ToCP	2013	Czech Republic	Homes	30	N.D	0.25	-	0	Vykoukalová et al.,2017
78-32-0	TpCP	2013	Czech Republic	Homes	30	0	2.5	-	0.2	Vykoukalová et al.,2017
2528-38-3	TPEP	2010–2011	Portugal	Homes	28	<0.00028	0.03	0	0	Coelho et al.,2016
		2013	UK	Homes	10	18.33	1010	152.69	64.55	Kademoglou et al.,2017
		2013–2014	Norway	Homes	10	1	33.89	5.83	1.96	Kademoglou et al.,2017
115-86-6	TPHP	2008	Belgium	Homes (floor)	33	0.04	29.8	2.02	0.5	Van den Eede et al.,2011
		2008	Belgium	Shop	15	0.15	34.2	4.7	1.97	Van den Eede et al.,2011
		2008	Danmark (Odense)	Daycare centers (shelves,ledges,windows sills)	151	<0.94	210	-	2	Langer et al.,2016
		2008	Danmark (Odense)	Homes (children bedroom's,shelves,ledges,windows sills)	497	<0.94	91	-	<0.94	Langer et al.,2016
		2008	Swenden (Stockolm)	Homes	62	0.68	38	-	4.3	Luongo and Östman.,2016
		2010	Roumania (Lasi)	Homes	47	<0.02	22.6	1.6	0.5	Dirtu et al.,2012
		2010	Roumania (Lasi)	Homes	47	<0.01	0.04	<0.01	<0.01	Dirtu et al.,2012
		2010–2011	Germany		10	0.47	4.8	2.5	-	Brommer et al.,2012
		2010–2011	Germany	Cars	12	0.5	11	3	2.3	Brommer et al.,2012
		2010–2011	Portugal	Homes	28	0.11	5.2	1	0.66	Coelho et al.,2016
		2011–2012	Germany	Daycare centers	63	<0.30	64.5	2.56	0.5	Fromme et al.,2014
		2011–2012	Germany	Cars	19	0.33	11	-	1.8	Harrad et al.,2016
		2011–2012	Germany	Homes (living rooms)	22	0.07	18	-	0.23	Harrad et al.,2016
		2011–2012	Germany	Offices	25	0.2	8.8	-	1.5	Harrad et al.,2016
		2011–2012	UK (West Midlands)	Cars	21	0.27	170	15	3.3	Brommer and Harrad.,2015
		2011–2012	UK (West Midlands)	Classroom	28	0.22	90	12	4.1	Brommer and Harrad.,2015
		2011–2012	UK (West Midlands)	Homes (living room)	32	0.49	110	10	3.3	Brommer and Harrad.,2015
		2011–2012	UK (West Midlands)	Offices	61	0.56	50	8.2	4.3	Brommer and Harrad.,2015
		2012	Norway	Homes	48	1.21	4.85	1.24	0.98	Cequier et al.,2014
		2013	UK	Homes	10	0.19	9.55	2.74	1.51	Kademoglou et al.,2017
		2013–2014	Norway	Homes	10	0.2	2.92	0.93	0.83	Kademoglou et al.,2017
		2013–2014	Norway (Oslo)	Homes (floor)	61	0.16	276	-	0.72	Xu et al.,2016
		2013–2014	Norway (Oslo)	Homes (surface)	61	0.33	956	-	1.23	Xu et al.,2016
		2013–2015	Netherlands	Homes (playroom)	50	0.19	9.86	-	0.55	Sugeng et al.2018
		2015	Germany (Rhine/Main area)	Cars	11	0.72	74	9.2	2.2	Zhou et al.,2017
		2015	Germany (Rhine/Main area)	Homes	15	0.48	23	3.6	1.2	Zhou et al.,2017
		2015	Germany (Rhine/Main area)	Offices	11	0.77	9.2	3.3	2.9	Zhou et al.,2017
		2016	The netherlands	Homes (on top of electronics)	11	0.29	45.33	10.35	9.79	Björnsdotter et al., 2018
		2016	The netherlands	Homes and Offices	12	0.17	12.85	3.07	1.44	Björnsdotter et al., 2018
		2017	Spain	Cars	15	0.76	142.46	18.31	4.44	Björnsdotter et al., 2018
		2017	Spain	Homes (on top of electronics)	13	1.27	2.62	59	2.42	Björnsdotter et al., 2018

		-	Greece	Cars	25	0.02	5.12	0.86	0.30 *	Christia et al.,2018
		-	Sweden (Stockolm)	Daycare center	10	0.3	17	3.5	1.9	Bergh et al.,2011
		-	Sweden (Stockolm)	Homes	10	0.1	4.2	1.6	1.2	Bergh et al.,2011
		-	Sweden (Stockolm)	Offices	10	0.9	32	8.8	5.3	Bergh et al.,2011
513-08-6	TPP	2008	Swenden (Stockolm)	Homes	62	<0.01	1.6	-	<0.01	Luongo and Östman.,2016
		2010–2011	Portugal	Homes	28	<0.86	10	3	2.4	Coelho et al.,2016
		2013	UK	Homes	10	<0.03	<0.03	<0.03	<0.03	Kademoglou et al.,2017
		2013–2014	Norway	Homes	10	<0.03	<0.03	<0.03	<0.03	Kademoglou et al.,2017
25653-16-1	TXP	2013	UK	Homes	10	<0.0011	0.54	0.08	0.03	Kademoglou et al.,2017
		2013	Czech Republic	Homes	30	N.D	N.D	-	N.D	Vykoukalová et al.,2017
		2013–2014	Norway	Homes	10	0	0.11	0.03	0.01	Kademoglou et al.,2017

-: information not available; N.D.: not detected; *: median calculated with detected measures LOD : limit of detection.

Supplementary Table S3. Concentrations of OPFRs in indoor air (ng/m3), Europe, 2000-2018.

CAS Number	Abbreviation	Period	Country	Sampling Location	Sample Size	Min	Max	Mean	Median	References
1241-94-7	EHDPP	2006–2007	Sweden (Stockolm)	Homes	169	N.D	24	5.6	<5.5	Bergh C et al.,2011
		2008	Sweden (Stockolm)	Homes	62	N.D	3.9	-	N.D	Luongo and Östman.,2016
		2012	Norway	Homes	47	-	3.51	0.489	0.119	Cequier et al.,2014
		2013	Czech Republic	Homes	29	0.193	1.09	-	0.375	Vykoukalová et al.,2017
		-	Sweden (Stockolm)	Homes	10	N.D	DET	N.D	N.D	Bergh et al.,2011
		-	Sweden (Stockolm)	Day care center	10	N.D	2.2	0.4	N.D	Bergh et al.,2011
		-	Sweden (Stockolm)	Office	10	N.D	14	1.8	N.D	Bergh et al.,2011
		2013–2014	Norway (Oslo)	Homes	61	<0.6	8	-	<0.6	Xu et al., 2016
		2014–2015	Sweden (Stockolm)	Office	23	<0.04	12	3.8	3.6	Wong et al., 2018
78-51-3	TBOEP	2006–2007	Sweden (Stockolm)	Homes	169	N.D	86	<31	<31	Bergh C et al.,2011
		2008	Sweden (Stockolm)	Homes	62	N.D	10	-	N.D	Luongo and Östman.,2016
		2012	Norway	Homes	47	-	18.2	1.45	0.598	Cequier et al.,2014
		2015	Germany (Rhine/Main area)	Offices	11	<0.68	5.33	0.79	<0.68	Zhou et al.,2017
		2015	Germany (Rhine/Main area)	School	12	<0.68	17.51	1.77	<0.68	Zhou et al.,2017
78-33-1	TBPP	2013	Czech Republic	Homes	29	N.D	0.001	-	0.001	Vykoukalová et al.,2017
115-96-8	TCEP	2006–2007	Sweden (Stockolm)	Homes	169	N.D	230	10	4	Bergh C et al.,2011
		2008	Sweden (Stockolm)	Homes	62	N.D	223	-	3.9	Luongo and Östman.,2016
		2012	Norway	Homes	47	-	10.1	3.23	2.25	Cequier et al.,2014
		2013	Czech Republic	Homes	29	0.781	36.4	-	2.96	Vykoukalová et al.,2017
		2015	Germany (Rhine/Main area)	Offices	11	<0.75	5.69	1.01	0.56	Zhou et al.,2017
		2015	Germany (Rhine/Main area)	Schools	12	<0.75	4.65	0.52	<0.75	Zhou et al.,2017
		-	Sweden (Stockolm)	Homes	10	N.D	28	8.3	4.8	Bergh et al.,2011
		-	Sweden (Stockolm)	Day care center	10	7.8	230	47	25	Bergh et al.,2011
		-	Sweden (Stockolm)	Office	10	N.D	140	21	10	Bergh et al.,2011
		2011–2012	Germany	Daycare centers	63	<2	33	2.2	<2	Fromme et al.,2014
		2013–2014	Norway (Oslo)	Homes	61	<0.9	76	-	3	Xu et al.,2016
		2014–2015	Sweden (Stockolm)	Office	23	6.7	20	11	10.9	Wong et al., 2018

		2015–2017	Sweden	Preschool	12	<2.2	<2.2	<2.2	<2.2	Persson et al.2018
		2015–2017	Sweden	Preschool	16	<2.2	<2.2	<2.2	<2.2	Persson et al.2018
		2015–2017	Sweden	Preschool	12	<2.2	<2.2	<2.2	<2.2	Persson et al.2018
		2015–2017	Sweden	Preschool	16	<2.2	<2.2	<2.2	<2.2	Persson et al.2018
13674-84-5	TCIPP	2006–2007	Sweden (Stockolm)	Homes	169	<0.5	1200	59	14	Bergh C et al.,2011
		2008	Sweden (Stockolm)	Homes	62	1.3	1179	-	19	Luongo and Östman.,2016
		2012	Norway	Homes	47	-	462	83.1	42.3	Cequier et al.,2014
		2013	Czech Republic	Homes	29	3.62	139	-	16.4	Vykoukalová et al.,2017
		2015	Germany (Rhine/Main area)	Offices	11	4.17	496.9	80.28	33.89	Zhou et al.,2017
		2015	Germany (Rhine/Main area)	Schools	12	4.14	68.83	17.53	9.99	Zhou et al.,2017
		-	Sweden (Stockolm)	Homes	10	2.4	64	15	5.6	Bergh et al.,2011
		-	Sweden (Stockolm)	Day care center	10	1.3	72	19	8.4	Bergh et al.,2011
		-	Sweden (Stockolm)	Office	10	16	240	110	100	Bergh et al.,2011
		2011–2012	Germany	Daycare centers	63	<2	45	4.1	2.7	Fromme et al.,2014
		2013–2014	Norway (Oslo)	Homes	61	<1.2	987	-	128	Xu et al., 2017
		2014–2015	Sweden (Stockolm)	Office	23	85	1900	480	330	Wong et al., 2018
		2015–2017	Sweden	Preschool	12	<14	34	<14	<14	Persson et al.2018
		2015–2017	Sweden	Preschool	16	<14	51	<14	<14	Persson et al.2018
		2015–2017	Sweden	Preschool	12	<14	93	21	<14	Persson et al.2018
		2015–2017	Sweden	Preschool	16	<14	<14	<14	<14	Persson et al.2018
13674-87-8	TDCIPP	2012	Norway	Homes	47	-	10.6	0.394	0.084	Cequier et al.,2014
		2013	Czech Republic	Homes	29	0.228	0.572	-	0.311	Vykoukalová et al.,2017
		2015	Germany (Rhine/Main area)	Offices	11	<0.10	2.89	0.57	0.42	Zhou et al.,2017
		2015	Germany (Rhine/Main area)	Schools	12	<0.10	29.86	2.99	<0.10	Zhou et al.,2017
		-	Sweden (Stockolm)	Homes	10	N.D	17	3.1	N.D	Bergh et al.,2011
		-	Sweden (Stockolm)	Day care center	10	N.D	30	6.7	N.D	Bergh et al.,2011
		-	Sweden (Stockolm)	Office	10	N.D	73	24	28	Bergh et al.,2011
		2014–2015	Sweden (Stockolm)	Office	23	N.D	-	N.D	-	Wong et al., 2018
		2015–2017	Sweden	Preschool	12	<11	<11	<11	<11	Persson et al.2018
		2015–2017	Sweden	Preschool	16	<11	<11	<11	<11	Persson et al.2018
		2015–2017	Sweden	Preschool	12	<11	<11	<11	<11	Persson et al.2018
		2015–2017	Sweden	Preschool	16	<11	<11	<11	<11	Persson et al.2018
78-42-2	TEHP	2013	Czech Republic	Homes	29	0.02	0.163	-	0.037	Vykoukalová et al.,2017
		2015	Germany (Rhine/Main area)	Offices	11	<0.06	0.87	0.16	<0.06	Zhou et al.,2017
		2015	Germany (Rhine/Main area)	School	12	<0.06	17.51	1.77	<0.06	Zhou et al.,2017
		-	Sweden (Stockolm)	Homes	10	N.D	N.D	N.D	N.D	Bergh et al.,2011
		-	Sweden (Stockolm)	Day care center	10	N.D	N.D	N.D	N.D	Bergh et al.,2011
		-	Sweden (Stockolm)	Office	10	N.D	N.D	N.D	N.D	Bergh et al.,2011
		2013–2014	Norway (Oslo)	Homes	61	<4.6	42	-	<4.6	Xu et al., 2016
		2014–2015	Sweden (Stockholm)	Office	23	0.08	0.64	0.24	0.19	Wong et al., 2018
78-40-0	TEP	2006–2007	Sweden (Stockolm)	Homes	169	<0.18	300	14	4	Bergh C. et al.,2011
		2008	Sweden (Stockolm)	Homes	62	0.84	297	-	5.3	Luongo and Östman.,2016
		2015	Germany (Rhine/Main area)	Offices	11	<0.32	18.34	1.85	<0.32	Zhou et al.,2017

		2015	Germany (Rhine/Main area)	School	12	<0.32	27.13	2.5	<0.32	Zhou et al.,2017
		2015–2017	Sweden	Preschool	12	<17	<17	<17	<17	Persson et al.2018
		2015–2017	Sweden	Preschool	16	<17	<17	<17	<17	Persson et al.2018
		2015–2017	Sweden	Preschool	12	<17	<17	<17	<17	Persson et al.2018
		2015–2017	Sweden	Preschool	16	<17	25	<17	<17	Persson et al.2018
126-71-6	TIBP	2006–2007	Sweden (Stockolm)	Homes	169	2	54	11	9	Bergh C. et al.,2011
		2008	Sweden (Stockolm)	Homes	62	2.4	53	-	8.9	Luongo and Östman.,2016
		2015	Germany (Rhine/Main area)	Offices	11	0.77	22.37	6.65	5.65	Zhou et al.,2017
		2015	Germany (Rhine/Main area)	School	12	1.59	36.78	12.76	8.56	Zhou et al.,2017
		-	Sweden (Stockolm)	Homes	10	3	66	18	13	Bergh et al.,2011
		-	Sweden (Stockolm)	Day care center	10	N.D	63	12	7.2	Bergh et al.,2011
		-	Sweden (Stockolm)	Office	10	4.4	13	7.8	7.3	Bergh et al.,2011
64532-95-2	TiPPP	2013	Czech Republic	Homes	29	0.003	0.023	-	0.005	Vykoukalová et al.,2017
		2013–2014	Norway (Oslo)	Homes	61	<1.5	<1.5	-	<1.5	Xu et al., 2016
1330-78-5	TMPP	2012	Norway	Homes	47	<MLD	0.644	0.08	<MLD	Cequier et al.,2014
126-73-8	TNBP	2006-2007	Sweden (Stockolm)	Homes	169	2.5	370	20	11	Bergh C et al.,2011
		2008	Sweden (Stockolm)	Homes	62	2.5	94	-	13	Luongo and Östman.,2016
		2012	Norway	Homes	47	-	124	9.66	5.09	Cequier et al.,2014
		2013	Czech Republic	Homes	29	0.745	33.1	-	2.34	Vykoukalová et al.,2017
		2015	Germany (Rhine/Main area)	Offices	11	0.29	112.1	18.5	5.85	Zhou et al.,2017
		2015	Germany (Rhine/Main area)	School	12	<0.05	25.34	4.25	2.78	Zhou et al.,2017
		-	Sweden (Stockolm)	Homes	10	3.5	45	16	9.1	Bergh et al.,2011
		-	Sweden (Stockolm)	Day care center	10	3.7	320	61	18	Bergh et al.,2011
		-	Sweden (Stockolm)	Office	10	N.D	100	21	2.3	Bergh et al.,2011
		2011–2012	Germany	Daycare centers	63	<2	1279	154	49	Fromme et al.,2014
		2011–2012	Germany	Daycare centers	63	<2	80	4	2.2	Fromme et al.,2014
		2013–2014	Norway (Oslo)	Homes	61	<2.7	119	-	14	Xu et al.,2016
		2014–2015	Sweden (Stockolm)	Office	23	0.92	2.1	1.2	1.2	Wong et al., 2018
		2014–2015	Sweden (Stockolm)	Office	23	0.9	4.3	1.8	1.3	Wong et al., 2018
		2015–2017	Sweden	Preschool	12	<19	<19	<19	<19	Persson et al.2018
		2015–2017	Sweden	Preschool	16	<19	82	<19	<19	Persson et al.2018
		2015–2017	Sweden	Preschool	12	<19	21	<19	<19	Persson et al.2018
		2015–2017	Sweden	Preschool	16	<19	150	36	<19	Persson et al.2018
115-86-6	TPHP	2006–2007	Sweden (Stockolm)	Homes	169	N.D	25	3.6	<3.1	Bergh C et al.,2011
		2008	Sweden	Homes	62	N.D	25	-	N.D	Luongo and Östman.,2016
		2012	Norway	Homes	47	-	1.65	0.375	0.258	Cequier et al.,2014
		2013	Czech Republic	Homes	29	0.304	0.91	-	0.592	Vykoukalová et al.,2017
		-	Sweden (Stockolm)	Day care center	10	N.D	0.9	0.1	N.D	Bergh et al.,2011
		-	Sweden (Stockolm)	Homes	10	N.D	0.8	0.2	N.D	Bergh et al.,2011
		-	Sweden (Stockolm)	Office	10	N.D	2.7	0.6	N.D	Bergh et al.,2011
		2013–2014	Norway (Oslo)	Homes	61	<0.3	9	-	1	Xu et al., 2016
		2014–2015	Sweden (Stockolm)	Office	23	0.16	0.67	0.31	0.29	Wong et al., 2018
		2015–2017	Sweden	Preschool	12	<2.6	6.7	<2.6	<2.6	Persson et al.2018

		2015–2017	Sweden	Preschool	16	<2.6	56	6.5	2.6	Persson et al.2018
		2015–2017	Sweden	Preschool	12	<2.6	4.8	<2.6	<2.6	Persson et al.2018
		2015–2017	Sweden	Preschool	16	<2.6	33	8.2	3.5	Persson et al.2018
513-08-6	TPP	2015	Germany(Rhine/Main area)	Offices	11	<0.16	0.64	0.16	<0.16	Zhou et al.,2017
		2015	Germany(Rhine/Main area)	School	12	-	-	<0.16	<0.16	Zhou et al.,2017
		2015–2017	Sweden	Preschool	12	<22	<22	<22	<22	Persson et al.2018
		2015–2017	Sweden	Preschool	16	<22	30	<22	<22	Persson et al.2018
		2015–2017	Sweden	Preschool	12	<22	<22	<22	<22	Persson et al.2018
		2015–2017	Sweden	Preschool	16	<22	23	<22	<22	Persson et al.2018
25653-16-1	TXP	2013	Czech Republic	Homes	29	N.D	N.D	-	N.D	Vykoukalová et al.,2017

-: information not avavailable; N.D.: not detected; DET: detected; MLD: method limit detection.

Supplementary Table S4. Biological measurment of exposure, Europe, 2000 -2019.

Matrix	Metabolites	Name	Country	Population	Size	Sampling Year	Min	Max	Mean	Median	References
Urine (ng/ml)	BBOEP	TBOEP	Norway	Mother	42	-	<0.6	-	<0.6	<0.6	Cequier et al.,2014
	BBOEP	TBOEP	Norway	Children (6-12 years)	42	-	<0.6	-	<0.6	<0.6	Cequier et al.,2014
	BBOEP	TBOEP	Norway	Mother	244	2012	<0.18	0.29	<0.18	<0.18	Cequier et al.,2015
	BBOEP	TBOEP	Norway	Children (6-12 years)	112	2012	<0.18	1	0.19	<0.18	Cequier et al.,2015
	BBOEP	TBOEP	Belgium	Adult (23 men and 36 women)	59	-	-	7	-	-	Van den Eede et al.,2013
	BBOEP	TBOEP	Germany	Children (20-80 months)	54	2011–2012	-	-	0.36	0.16	Völkel et al., 2018
	BBOEHEP	TBOEP	Germany	Children (20-80 months)	54	2011–2012	-	-	0.33	0.6	Völkel et al., 2018
	DBEP	TBOEP	Germany	Children (22-80 months)	312	2011–2012	<0.015	24.9	3.2	2	Fromme et al.,2014
	BCEP	TCEP	Germany	General population (11-68 years)	30	2008	<0.1	27.5	-	<0.1	Schindler et al.,2009
	BCEP	TCEP	Belgium	Adult (23 men and 36 women)	59	-	-	9.5	-	-	Van den Eede et al.,2013
	DCEP	TCEP	Germany	Children (22-80 months)	312	2011–2012	<0.1	13.1	0.4	0.2	Fromme et al.,2014
	BCPP	TCIPP	Germany	General population (11-68 years)	25	2008	-	0.85	-	<0.25	Schindler et al.,2009
	BCPP	TCIPP	Belgium	Adult (23 men and 36 women)	59	-	-	6.2	-	-	Van den Eede et al.,2013
	DCPP	TCIPP	Germany	Children (22-80 months)	312	2011–2012	<0.2	8.4	0.2	<0.2	Fromme et al.,2014
	BDCIPP	TDCIPP	Norway	Mother	42		-	-	0.13 **	<0.4	Cequier et al.,2014
	BDCIPP	TDCIPP	Norway	Children (6-12 years)	42	-	-	-	0.2 **	-	Cequier et al.,2014
	BDCIPP	TDCIPP	Norway	Mother	244	2012	-	2	0.2	0.12	Cequier et al.,2015
	BDCIPP	TDCIPP	Norway	Children (6-12 years)	112	2012	-	3.7	0.33	0.23	Cequier et al.,2015
	BDCIPP	TDCIPP	Belgium	Adult (23 men and 36 women)	59	-	-	15	-	-	Van den Eede et al.,2013
	DEHP	TEHP	Germany	Men and women (14-85 years)	19	-	-		-	1.4	Reemtsma et al.,2011
	DEP	TEP	Germany	Men and women (14-85 years)	19	-	-		-	3.2	Reemtsma et al.,2011
	DmCP	TmCP	Germany	Children (22-80 months)	312	ND	N.D	N.D	N.D	N.D	Fromme et al.,2014
	DmCP	TmCP	Germany	General population (11-68 years)	30	2008	-	<0.5	-	<0.5	Schindler et al.,2009
	DBP	TNBP	Germany	General population (11-68 years)	25	2008	-	0.26	-	<0.25	Schindler et al.,2009
	DBP	TNBP	Belgium	Adult (23 men and 36 women)	59	-	-	3.5	-	-	Van den Eede et al.,2013
	DNBP	TNBP	Norway	Mother	42	-	<0.4	-	<0.4	<0.4	Cequier et al.,2014
	DNBP	TNBP	Norway	Children (6-12 years)	42	-	<0.4	-	<0.4	<0.4	Cequier et al.,2014
	DNBP	TNBP	Norway	Mother	244	2012	<0.12	0.49	<0.12	<0.12	Cequier et al.,2015
	DNBP	TNBP	Norway	Children (6-12 years)	112	2012	<0.12	0.87	<0.12	<0.12	Cequier et al.,2015
	DNBP	TNBP	Germany	Children (22-80 months)	312	2011–2012	<0.1	6.6	0.3	0.2	Fromme et al.,2014
	DoCP	ToCP	Germany	Children (22-80 months)	312	ND	N.D	N.D	N.D	N.D	Fromme et al.,2014
	DpCP	TpCP	Germany	Children (22-80 months)	312	ND	N.D	N.D	N.D	N.D	Fromme et al.,2014
	DpCP	TpCP	Germany	General population (11-68 years)	30	2008	-	<0.5	-	<0.5	Schindler et al.,2009

	DPHP	TPHP	Norway	Mother	42	-	-	-	1.1 **	-	Cequier et al.,2014
	DPHP	TPHP	Norway	Children (6-12 years)	42	-	-	-	0.57 **	-	Cequier et al.,2014
	DPHP	TPHP	Norway	Mother	244	2012	-	60	1.2	0.51	Cequier et al.,2015
	DPHP	TPHP	Norway	Children (6-12 years)	112	2012	-	135	3.2	1.1	Cequier et al.,2015
	DPHP	TPHP	Germany	Children (22-80 months)	312	2011–2012	<0.15	23.2	1.4	0.8	Fromme et al.,2014
	DPHP	TPHP	Sweden	Children (4 years)	113		0.45	35	-	1.8	Larsonn et al.,2014
	DPHP	TPHP	Germany	Men and women (14-85 years)	19	-	-		-	1.3	Reemtsma et al.,2011
	DPHP	TPHP	Belgium	Adult (23 men and 36 women)	59	-	-	13	-	-	Van den Eede et al.,2013
	DPP	TPHP	Germany	General population (11-68 years)	30	2008	-	5.47	-	0.52	Schindler et al.,2009
Hair (ng/g)		EHDPP	Norway	Mother	48	2012	5	265	-	27	Kucharska et al.,2015
		EHDPP	Norway	Children	54	2012	2	346	-	21	Kucharska et al.,2015
		TBOEP	Norway	Mother	48	2012	14	1253	-	65	Kucharska et al.,2015
		TBOEP	Norway	Children	54	2012	34	2411	-	318	Kucharska et al.,2015
		TCEP	Norway	Mother	48	2012	<33	163	-	72	Kucharska et al.,2015
		TCEP	Norway	Children	54	2012	<33	118	-	59	Kucharska et al.,2015
		TDCIPP	Norway	Mother	48	2012	<9	3744	-	30	Kucharska et al.,2015
		TDCIPP	Norway	Children	54	2012	<9	2698	-	31	Kucharska et al.,2015
		TMPP	Norway	Mother	48	2012	<2	134	-	8	Kucharska et al.,2015
		TMPP	Norway	Children	54	2012	<2	74	-	8	Kucharska et al.,2015
		TNBP	Norway	Mother	48	2012	5	672	-	22	Kucharska et al.,2015
		TNBP	Norway	Children	54	2012	3	150	-	11	Kucharska et al.,2015
		TPHP	Norway	Mother	48	2012	5	1256	-	52	Kucharska et al.,2015
		TPHP	Norway	Children	54	2012	6	363	-	63	Kucharska et al.,2015
Milk (ng/g of lipid weight)		TBOEP	Spain	Breast milk	20	2015	<0.03	14.8	14.8	14.8	Beser et al.,2019
		TCEP	Spain	Breast milk	20	2015	<0.02	-	-	-	Beser et al.,2019
		TCIPP	Spain	Breast milk	20	2015	<0.13	12.5	12.5	12.5	Beser et al.,2019
		TEP	Spain	Breast milk	20	2015	<0.02	7.8	4	3.4	Beser et al.,2019
		TIPP	Spain	Breast milk	20	2015	<1.9	-	-	-	Beser et al.,2019
		TMP	Spain	Breast milk	20	2015	<0.32	19	19	19	Beser et al.,2019
		TNBP	Spain	Breast milk	20	2015	<0.03	10.9	10.9	10.9	Beser et al.,2019
		TnPrP	Spain	Breast milk	20	2015	<1.9	-	-	-	Beser et al.,2019
		TPHP	Spain	Breast milk	20	2015	<0.12	17.3	12.3	9.9	Beser et al.,2019
		BdPhP	Sweden	Breast milk	286	1997–2007	N.D	-	-	N.D	Sundkvist et al.,2010
		dBPhP	Sweden	Breast milk	286	1997–2007	N.D	-	-	N.D	Sundkvist et al.,2010
		EHDPP	Sweden	Breast milk	286	1997–2007	3.5	13		-6.5	Sundkvist et al.,2010
		TBOEP	Sweden	Breast milk	286	1997–2007	N.D	63		-4.7	Sundkvist et al.,2010
		TCEP	Sweden	Breast milk	286	1997–2007	2.1	8.2		-4.9	Sundkvist et al.,2010
		TCIPP	Sweden	Breast milk	286	1997–2007	22	82		-45	Sundkvist et al.,2010
		TDCIPP	Sweden	Breast milk	286	1997–2007	1.6	5.6		-4.3	Sundkvist et al.,2010
		TMPP	Sweden	Breast milk	286	1997–2007	N.D	3.7		-0.8	Sundkvist et al.,2010
		TNBP	Sweden	Breast milk	286	1997–2007	3.5	13		-12	Sundkvist et al.,2010
		ToCP	Sweden	Breast milk	286	1997–2007	N.D	-	-	N.D	Sundkvist et al.,2010
		TPHP	Sweden	Breast milk	286	1997–2007	3.2	11		-8.5	Sundkvist et al.,2010

-: information no available; N.D.:not detected; **: mean calcultated with detected measures.
